# The emergence of the Medical University of Vienna 20 years ago

**DOI:** 10.1007/s00508-024-02334-4

**Published:** 2024-03-08

**Authors:** Wolfgang Schütz, Markus Grimm

**Affiliations:** 1 Center for Physiology and Pharmacology, Medical University of Vienna, Schwarzspanierstraße 17A, 1090 Vienna, Austria; 2 Legal Affairs and Compliance, Medical University of Vienna, Spitalgasse 23, 1090 Vienna, Austria

**Keywords:** Austria, Public university, Autonomy of universities, Tenured positions, University-affiliated hospitals

## Abstract

At the beginning of the 2000s the Austrian public universities were characterized by staffing rigidities, little competitive research, outdated study curricula and free access to all fields of study, the latter combined with high dropout rates and long study durations. As a countermeasure the universities were granted full legal capacity. For new employees the status of civil servants was herewith cancelled and, being now subject to the Employment Act, tenured employments for anyone who wanted to stay at the university were no longer possible. Medical faculties always had special provisions which would be difficult to reconcile with the full legal capacity of the universities: (i) the requirements of the hospitals affiliated to universities for research and teaching in addition to patient care had to be reimbursed to the Austrian federal states maintaining the hospitals, (ii) the physicians of university-affiliated hospitals were largely employed by the respective university and (iii) to ensure financing of clinical research and teaching at the hospital, the medical faculties received a budget separated from the rest of the university. As it was neither politically possible nor foreseeable that universities would be able to form a subcorporation with the affiliated hospital (integration model) or at least a close cooperation with the hospital if that has legal capacity per se (cooperation model), the necessary budgetary sovereignty of the medical faculties could only be guaranteed by their transition to medical universities. Nonetheless, reservations about this spin-off of medicine were enormous, but quickly fell silent, as the newly established medical universities maintained close cooperations with their parent as well as other universities and achieved, for Austrian standards, favorable positions in international rankings.

In 2004, the publicly funded Austrian universities became legal entities of public law, and, in this process the three previously existing medical faculties of the Universities of Vienna, Graz and Innsbruck became independent universities. How did this come about?

At the beginning of the 2000s, the Austrian public universities were at a crossroads, namely in the sense of continuing as it is or embedding them in a completely new legal form in which they would be given full responsibility for independent action. The international position of the universities, which until then had been subordinate to the Austrian Federation, was miserable; the professors were only interested in their own institute or, in the clinical field, in their own clinical department, but in no way in the prosperity of their university [1]. The representatives of the academic mid-level staff were, like the professors, civil servants, and thus consistently tenured employees. Hence, there was only little room for next generation scientists. Students were admitted on a free access basis, so the number of students at the beginning of the studies was exorbitantly high, the dropout rate accordingly just as high and any modernization of study programs failed due to missing parliamentary majorities: fields of study, such as medicine, were designated by federal law [2] and regulated in detail by a ministerial order according to a general law on higher education [3]. In contrast, extramural research institutions where no students had to be supervised flourished, i.e. there were no undergraduate courses, otherwise a compulsatory activity that was considered annoying and time-consuming for reasons mentioned above: (i) in 1985, the Research Institute of Molecular Pathology was founded as a joint venture of the companies Boehringer Ingelheim and Genentech [4]. It was settled on the former Hornyphon premises in Vienna’s 3rd district and formed the core of today’s Vienna Biocenter [5]. (ii) During the 1990s, the last decade before the universities became autonomous, the number of institutes founded in the Austrian Academy of Sciences became exorbitantly high, doubling from 16 to 33 [6]. In the meantime, the number has declined and currently there are 26 institutes [7]. (iii) An initiative launched in 2002 to establish a university of excellence or an institute for top-level research, the working names at that time, was also successful resulting in today’s Institute for Science and Technology Austria (ISTA) [8], where only doctoral study programs were and are applied. Both the Academy of Sciences and the ISTA were publicly funded.

In this article, the European meaning for the term “Faculty” is used, namely to refer to a group of departments in a university or college that specializes in a particular subject, which is distinct from its American meaning that a faculty is determined by people who teach in a university, college or high school, or in one of its departments [9].

## Public universities in Austria and the medical faculties before 2004

In 1975, only with the votes of the Socialist Party, which held an absolute parliamentary majority at that time, the Austrian Parliament (the National Council) passed the University Organization Act, one of the most controversial and long-discussed laws in Austria at all after the end of the World War II [10]. The previously dominant position of the professors was severely weakened: before, the academic mid-level staff and students had no voting rights at all but now all bodies and committees had to be composed of only 50% professors and 25% each of mid-level staff and students, and, moreover, in institute and clinical department panels, where in particular the election of the department heads took place, professors, mid-level staff and students even received the same share of votes. Furthermore, the duration of the period as head of a department was limited to only 2 years. Only professors were allowed to be heads but a professor who wanted to become head therefore had to rely on the goodwill of all the academic staff and of the students.

Personal matters were duties of special faculty committees, which quite often decided on new employees against the will of the department head, whose right was limited only to propose candidates, and sometimes decided also against the will of the professors in the committees who, as mentioned above, did not have a majority. After 4 years, for physicians in residency training after 7 years (i.e., until board certification), the employees, previously civil servants on limited appointment, were taken on as civil servants with full and as a rule nonterminable appointments if suitable; however, in the opinion of the committees suitability (for de facto tenured positions) was almost always given. Although the Ministry for Science and Research was responsible for final decisions, it did not make use of its right to check whether the new civil servant candidate was needed at all. The ministry not only executed the will of the university’s personnel committees quasi as a rule, but even counteracted it in the few cases in which the committees indeed made a negative decision [11]. It was, therefore, foreseeable that soon there would be no more positions available for new scientific staff members and that the universities would be threatened with personnel petrification. Only then in 2002, under a right-wing government, namely a coalition between the People’s Party and the equipotent Freedom Party, was the emergency brake applied: Austria’s public universities became legal entities, were thus separated from federal sovereignty and had to constitute themselves under conditions of autonomy and self-administration [12], which also meant the abolition of public service for newly admitted staff members.

According to amendments of the University Organization Act from 1975 [10], the medical faculties had a special status within the universities due to the following circumstances [13–15]: (i) Austria is a federal republic with nine federal states. The federal government is responsible for the universities but, however, the state governments are responsible for the hospitals thus, for instance, the City of Vienna is responsible for the Vienna General Hospital, the hospital attached to the University of Vienna’s medical faculty. Its full staff physicians (more than 1700) all of whom are employed at the University of Vienna and thus in service of the Republic, but on the contrary, the nursing staff and administrative personnel are in service of the hospital holding company (which, in Vienna, is the municipality itself). Only about half of the physicians working in the university-attached state hospitals of Graz and Innsbruck are employed by the respective universities. (ii) As provision of healthcare is an official duty for these physicians it has to be carried out together with duties of research and teaching (triple track). By the way, the abovementioned staffing rigidity of Austrian universities attributable to an unchecked awarding of tenured positions was less pronounced among physicians at university hospitals because after completing their residency training, they found and find even higher income jobs outside the university, be it in private practice and/or in senior physician positions in hospitals. (iii) Most importantly, due to their healthcare duties the medical faculties received their own federally allocated budget, clearly separated from the rest of the University. There is a still valid special regulation not included in the University Organization Act but in the Federal Hospitals Act [16] which in essence dates back to 1920 [17]: direct payments have to be made by the Federal Government to the respective federal states as the financing authorities for their university-affiliated hospitals to cover the hospital’s needs arising from the operation of research and teaching in addition of patient care (additional clinical work, called “separation calculation” in Germany and Switzerland).

## 2004: public universities in Austria become legal entities

With the implementation of the Universities Act 2002 [12] the Austrian universities became legal entities in public law. Endowed with freedom from governmental regulation they are now allowed to operate in an entrepreneurial-like manner. In spite of the necessity of this reform step for strengthening the international reputation of Austrian universities, it is still lamented today that the much-vaunted participation of students and mid-level staff as a core element of the predecessor law was gone; however, real participation has never existed. Due to missing autonomy, participation was limited only to internal processes of less importance. With respect to essential resources, such as finances, personnel and buildings, but also to structure and organization, participation so far has been manifested only in requests of the universities to the Austrian Federation, represented by the Federal Minister responsible for science and research; however, these requests were often just pie in the sky, which the minister, primarily responsible for his own budget, only complied to a limited extent. In the context of the Universities Act 2002 the university, headed by the rector and a team of vice-rectors, is now responsible for its entire budget, personnel and structure and a university council acts as a supervisory board. The participation of the university members is now limited to tasks incumbent upon the senate: participation in the election of the rector, appointment of half of the members minus one of the university council, enacting and amending the statutes of the university, prescribing the curricula of studies and courses. However, in contrast to the lack of autonomy of the universities before, parity-based participation (the representatives of the professors make up only half of the members of the senate) now actually leads to concrete results and not merely to requests.

The universities are now also employers and have significantly increased possibilities and flexibility in the organization of working conditions and in personnel management. For the newly admitted staff, the Salaried Employees Act and a separate collective agreement apply [18]. The regulations of the Public Service Law continue to apply to the naturally decreasing number of employees who have still been admitted, due to the University Organization Act before [10], as civil servants. An essential core of the universities’ collective agreement is the establishment of a performance-oriented career model to attain a tenured position, the key points of which apply uniformly to all Austrian universities but give ample scope for maneuver to develop a university’s own career schemes depending on its location, requirements and general conditions. There is no right in getting a career position, but the fulfilment of the performance targets defined in a competitively awarded qualification agreement leads to a permanent, but nonetheless terminable, employment contract as an associate professor. Full professorships are subject to separate appointment procedures.

**Fig. 1:**
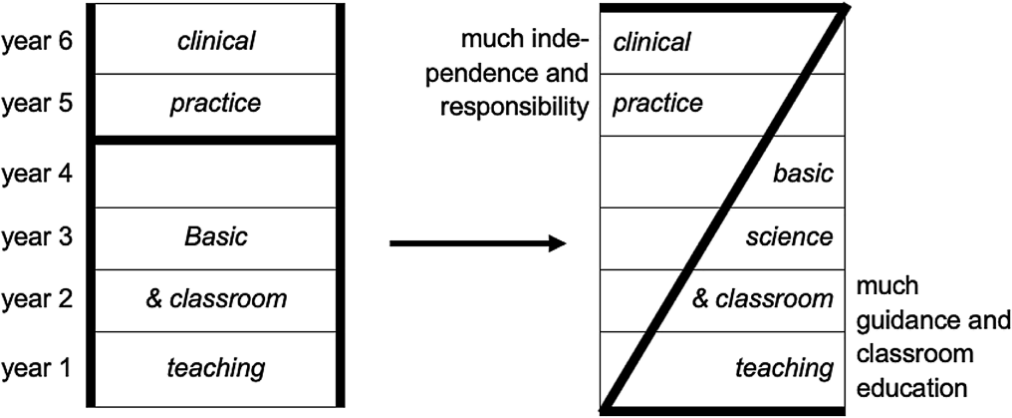
The traditional H‑shaped medical curriculum is being replaced by a Z-shaped curriculum model (from [21], reprinted by permission of Informa UK Limited, traded as Taylor & Francis Group, www.tandfonline.com)

The possibility to create their own study curricula was already given to the Austrian universities a few years before in 1997 [19] and the medical faculties of the three public universities made immediate use of it in order to finally dismiss a 100-year-old study law for medicine [20], which was only marginally modified in 1973 [2]. The strict adjoining sequence, first a lengthy preclinical then the clinical phase, was replaced by an integrated curriculum, where both phases are combined and taught together from the beginning (from H to Z model, Fig. 1; [21, 22]).

## The establishment of legally autonomous medical universities

Autonomous medical universities are uncommon in the international university scene. Nevertheless, many presidents of US universities with a medical school would be happy to be rid of this constantly money-demanding and resource-consuming institution [23]. In Europe, there are only two historically grown examples, the Karolinska Institute in Stockholm and the Hannover Medical School, both highly respected institutions with international reputation and both had and have an ideal structure for university medicine, from which Austria was and still is far away: the hospital belongs to the university. This version was not wanted by the responsible body financing public universities, namely the Austrian Federal Government because (i) of the high costs required for a hospital of the highest level of care, and (ii) inpatient healthcare in Austria in the first place is a responsibility of the federal states and not of the federation itself. The political decision, after a long discussion, to separate the medical faculties from their traditional university body in the form of medical universities was ultimately based on three reasons:

1. The size of the medical faculties and their share of total university costs*. *The size of the university medicine in Vienna, consisting of the preclinical institutes of the University of Vienna and the inpatient departments of the university-affiliated General Hospital, was and is unique in international comparison: on the one hand, the Vienna General Hospital as the largest hospital in Europe with 2206 beds and 1757 physicians as of 2003, all of them scientific staff members of the University of Vienna’s medical faculty and paid by the federal budget and on the other hand a total number of staff members of the University of Vienna’s medical faculty, including those of the preclinical area of 3198 [24]. Furthermore, up to 2005 there was unregulated access to medical studies with more than 2000 beginners every year, and only thereafter it was allowed by law to define a limited number of study places (with associated entrance test), namely 740, which is still extremely high from an international perspective. For comparison, the 155 US medical schools have on average of only 148 study places, and from all US medical schools together there actually are 21,000 medical graduates per year [25]. Standardized to the number of US inhabitants (= 333 million), this amounts to only one third of the 1735 medical graduates (year 2021/2022) from Austrian universities [26]! Table 1 shows the costs for the University of Vienna’s medical faculty in relation to the costs for the entire university for 2003, its last year before separation. It also shows the direct payments made by the Federal Government to the City of Vienna, as the maintaining body of the Vienna General Hospital, to cover the hospital’s needs arising from the common operation with research and teaching. The costs incurred by the Federal Government for university medicine in Vienna thus amounted to 58% of the costs incurred by the entire University of Vienna [27]. Similar criteria and ratios, with lower absolute figures, existed for the two other medical faculties at the university locations in Graz and Innsbruck.
2. Continuity of the existing special legal conditions. Even before their separation, medical faculties had a special budgetary status within their universities as the university physicians had to be involved in the healthcare duties of the associated university hospital but also the budget for consumables and investment in a university hospital was largely tied up in the sense of joint operation of healthcare, research and teaching. For this reason, the budget of a medical faculty could not be subjected to the discretion of the rector of an autonomous university if the faculty would belong to it, and in the past, therefore, the medical faculties in Austria were allocated their own budget, separate from the other faculties of the university.
3. University-affiliated hospitals in Austria are not legal entities per se*. *The first two reasons would not in themselves have been valid for the separation of a medical faculty from its university even if this university has full legal capacity (although the vast majority of the costs the university would have to allocate to its medical faculty, see Table 1, ended to speak in favor of a separation). In an international comparison, many universities either have outsourced their medical faculty, or at least its clinical part, into a subcorporation with the affiliated hospital (integration model) or form a close cooperation with the hospital if that has legal capacity per se (cooperation model) [28]. Thus, both partners must provide financial security for the joint operation of healthcare, research and teaching. Table 2 shows examples that already existed at that time.

**Table 1:** Federal expenditures (in million Euros) for university medicine in Vienna in 2003 (data from [24, 27])

	University of Vienna	Thereof its Medical Faculty
Expenses for the University of Vienna	417,150	187,367
Current expenses for additional clinical work^a^	94,213	94,213
Building and equipment expenses for additional clinical work^a^	36,863	36,863
*Total expenses*	*548,226*	*318,443*

^a^Direct federal payment to the City of Vienna as the maintaining body of the Vienna General Hospital for its additional needs arising from research and teaching operated simultaneously with patient care

**Table 2:** Structure models for university-affiliated hospitals (only valid for universities as legal entities)

Structure model	Examples	Medical faculty justified as medical university
Hospital belongs to the university	Karolinska Institute, Stockholm Hannover Medical School	Yes
Hospital and university form a subcorporation in public law (integration model)	Charité-University Medicine Berlin University Medical Center Hamburg-Eppendorf Amsterdam University Medical Center	No
Cooperation of two legal entities, university and hospital (cooperation model)	Heidelberg University Hospital LMU^a^ Hospital	No
Cooperation of the university and a hospital holding company^b^	Medical Universities in Austria^c^	Yes

^a^Ludwig Maximilian University of Munich

^b^In Austria 2004: Styrian Hospital Society (KAGES, Steiermärkische Krankenanstaltengesellschaft) and Tirol Kliniken; in Vienna the municipality by itself was and is the maintaining body of the Vienna General Hospital

^c^There are cooperation contracts in Vienna and Innsbruck only since 2016, a little earlier in Graz, but there were no contracts before

In Austria, none of the first three structural models shown in Table 2 were possible and still are not. Neither hospital affiliated to a public university in Austria, although hospitals of the highest level of care, was an independent legal entity, but belonged to a superordinate hospital holding company, namely the State Hospital of Graz to the Styrian Hospital Society (KAGES, Steiermärkische Krankenanstalten Gesellschaft), the State Hospital of Innsbruck to Tirol Kliniken, both limited liability companies, and the Vienna General Hospital had and still has, as most of the public Viennese hospitals, the City of Vienna itself as the maintaining body. None of the federal states and none of the aforementioned holding companies, already owned by the respective federal state, were willing to outsource its university hospital as an independent legal entity. Hence, every university with a medical faculty in Austria would have had no direct contractual partner, but always only a partially responsible partner, up to and including an entire federal state (the City of Vienna). It should also be added that universities and hospitals in Austria belong to different governmental authorities (universities to the federation, hospitals to the federal states) and are therefore financed differently, which is not the case in the other European countries. There is either, and largely, no federal structure or both universities and healthcare facilities are under the responsibility of the federal states, as is the case in Germany.

Moreover, at the time the medical faculties became autonomous in 2004, but also for many years afterwards, in Vienna until 2016, there was no cooperation contract at all between the Medical University and the maintaining body of the Vienna General Hospital, the City of Vienna. During those 12 years, only the legally prescribed federal payment to the City of Vienna and the other federal states for the additional needs of their hospital arising from the common operation with research and teaching was continued (see Table 1), but now allocated via the medical universities as a transitory item. The agreement concluded in 2016 between three partners, the Medical University, the City of Vienna and the Federal Government, stipulated, however, more intensive cooperation but is far away from an internationally established cooperation model or, let alone, integration model between a university and a legally independent hospital as is evident from Table 2. The Austrian Court of Audit, which audited the cooperation between the federation and the City of Vienna in 2012/2013 using the Vienna General Hospital as an example, merely suggested better cooperation [29] but most regrettably, no contribution to a sufficient cooperation model for medical universities in Austria with their university hospitals was made from this side either. Due to an apparently unrealizable internationally proven model of cooperation between university and hospital, the political decision at the time was that (i) the necessary budgetary sovereignty for university medicine could only be guaranteed with independent medical universities, and (ii) positioning in a loose cooperation with a hospital holding company only or—still aggravating—with a federal state as the City of Vienna, whose interests in research and teaching are expected to be limited and where many day to day decisions have to be made, was easier as a separate medical university than as part of a full university.

## The separation process

The separation of medicine from its parent university sometimes led to more heated discussions than the transition of public universities to independence itself. It was argued that medicine would lose touch with the other sciences, in which it would allegedly have been so well embedded, cooperation with the disciplines represented by the other faculties within the university would also be more difficult and therefore medicine as an independent academic institution would be reduced to the level of a simple college, there were no international examples of medical universities and if there only ones from the former socialist Eastern bloc countries, there would be a serious break with tradition and that an additional administrative apparatus would also have to be set up. Also, the medical faculties themselves did not agree on what they really wanted: that in Graz was overwhelmingly in favor of the separation, that in Innsbruck against, while in Vienna the opinion was 50-50.

As expected, the universities that hosted the medical faculties fully agreed that medicine must not go for the reasons mentioned above but the fear of a future loss of power if the universities’ largest faculty by far will get lost probably played the main role. In any case, the parent universities continued to fight against the foundation of separate medical universities long after the political decision on July 2002 had been made. In doing so, they had the public opinion on their side as the factual arguments cited in favor of the separation were too complex to be generally understood or got lost in the general emotion surrounding this issue. In addition, particularly in academic circles there was opposition to a government coalition in which the far-right Freedom Party was equally strongly represented as the People’s Party, and thus also opposition against the legislation associated with this government. The Universities Act 2002 was the last major law passed by this coalition: the vote on it in the Austrian National Council took place on 11 July 2002 [30], shortly thereafter collapse of the government took place and new elections were declared [31]. As a result of this diverse background, classical wars of roses broke out between the universities and their medical faculties, particularly at the University of Vienna and the University of Innsbruck, as the separation processes had to begin.

A splitting of assets and infrastructure facilities was necessary. Services that the parent university had previously also provided for medicine should, if possible, continue to be provided for both universities. Otherwise, if there were objective reasons for not doing so, such services would now have to be provided by the new medical university itself and therefore the funds saved by the parent university would also have to be transferred. This was intended to counter a major criticism of the separation of medicine, namely that it would be associated with increased administrative costs. One can imagine, or rather it was in the nature of the problem, that there was no enthusiasm of the parent university, which was against the separation, for such a splitting of assets. On the one hand, the medical faculties were aware that the parent university was much more experienced in administrative matters but on the other hand, they feared that the emotional sentiment directed against them would also affect the will of the employees in the central administration of the parent university to provide services with equal dedication for a now spun-off medical faculty. For this reason, despite long and numerous rounds of negotiations, joint services were limited to a small extent as was the transfer of funds to medicine for a reduced range of services at the parent university due to separation. In any case, the conclusion was that a cost neutral separation on an administrative basis was not possible, which, however, was largely and generally due to the spin-off of the Austrian universities from the federal administration. Regardless of this and shown in Table 3, the administrative costs of the medical universities are, in terms of staffing levels, significantly lower than those of all other public universities in Austria [32]. Nevertheless, the Medical University of Vienna was “endowed” in its opening balance with a negative equity of € 8.33 million by the Ministry responsible for science and research [33]. It was able to live with this.

**Table 3:** Full-time equivalents (*FTE*) for the administrative staff in active service, including staff covered by third-party and special funding for 2022 (data from [32])

	All universities	Medical University of Vienna
*Total FTE*	40,343	4,744
*FTE administration*	14,330	973
*Proportion of administration*	36%	21%

## Later reactions following the separation

Emotions gradually subsided after the University Act 2002 had come into force on 1 January 2004. Even Austria’s three major universities, which had lost their medical faculties, ultimately accepted the separation and focused on their own development and reputation. Both were also achieved through cooperation with their former medical faculties, and it could be seen that, at least in Vienna, cooperation became more diverse and intensive than cooperations had previously been within the University of Vienna between the medical faculty and others. Examples of structured cooperations defined by contract are the Max Perutz Laboratories founded in 2005 [34], the Institute for Ethics and Law in Medicine [35] and inter-university research clusters established in 2011 [36]; however, in 2011, the then Federal Minister of Science and Research, a classical philologist and himself previously Rector of the University of Innsbruck, took office for 2.5 years with his main aim of reintegrating medicine into its former universities [37]. As neither the medical universities nor their former parent universities had announced any intention to pursue this idea, the minister limited his plan to supplementary paragraphs in the Universities Act 2002 intending to generally facilitate the unification of universities and to enable the establishment of medical faculties [38]; however, both measures would have been possible without such amendment, leading many experts to question its purpose. Today, 11 years later, no universities have yet considered merging. Furthermore, the term “faculty” has been deliberately omitted from the University Act, as it should be left to the freedom of a university to establish faculties.

In the wake of this amendment, a faculty of medicine was established instead in 2014 at the Johannes Kepler University Linz at the insistence of the state of Upper Austria. Originally, there was the desire for a medical university comparable to the existing ones, but the state changed its request to a medical faculty within the Johannes Kepler University, which was easier to push through under this minister. As the associated hospital, the Kepler University Hospital, became a limited liability company [39], the cooperation between two legal entities, university and hospital, after all represents an internationally proven cooperation model (Table 2), which makes it possible to secure a separate budget, at least for clinical medicine, even within a legally responsible university having other (i.e., non-medical) faculties and schools as well. Hence, there was a significant difference to the university locations of Vienna, Graz and Innsbruck, where the university hospitals were not and are not separate legal entities, but belong to a superordinate hospital association, in Vienna even to an entire federal state.

For the Kepler University Hospital too, the Federal Government per se is responsible for cost replacement to Upper Austria as the federal state the hospital belongs to, concerning research and teaching requirements to identify and manage the additional clinical work (Table 1). This in its essence more than 100 years old regulation [17], albeit elevated to federal constitutional status, deserves to be abolished as soon as possible, because the calculation of such expenses and their reimbursement should be (i) a duty of the now autonomous universities, and (ii) the fund recipient should be the real cooperation partner, ideally a university-affiliated hospital with legal capacity, and not the federal state, who is not obliged to earmark this funding.

## Development of the medical universities in Austria

**Fig. 2:**
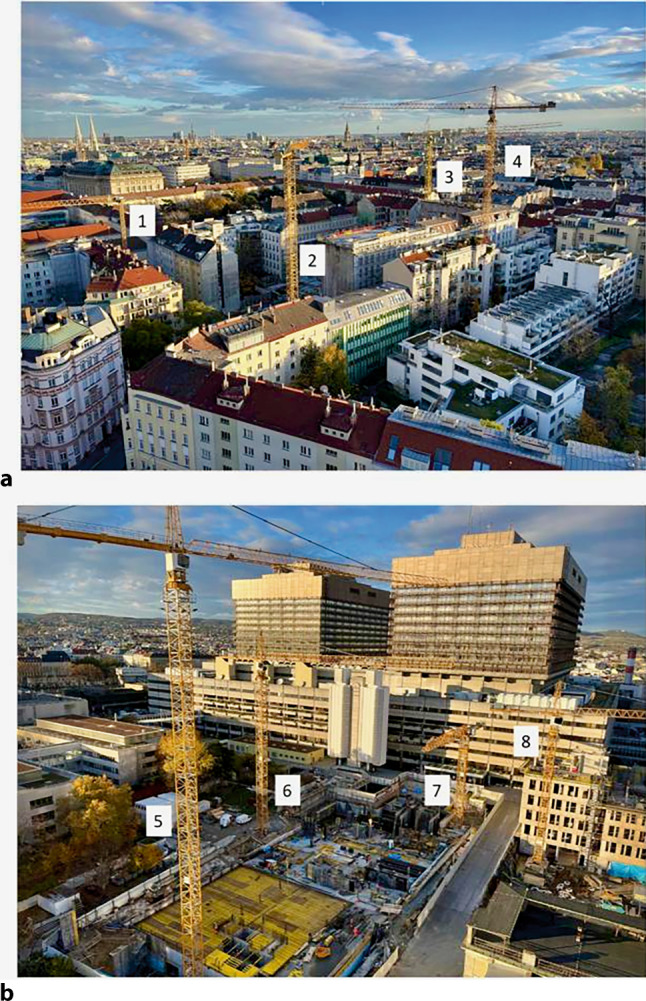
Construction sites within the area for the Medical University of Vienna. **a** the new campus building for nonclinical departments (cranes 1–4). **b** Eric Kandel Center for Precision Medicine (cranes 5–7) and the Center for Translational Research (crane 8). The core building of the Vienna General Hospital can be seen in the background (photograph from 20 November 2023). Photograph by courtesy of Markus Müller

In the international university ranking Times Higher Education (THE), yearly published over the last 13 years [40], the Medical University of Vienna has been 5 times among the top 100 in the “Clinical and Health” category (top 95 in 2024), including once, in 2014, among the top 50 universities worldwide. In the overall international university rankings, the three medical universities of Graz, Vienna and Innsbruck are consistently behind the University of Vienna, but ahead of all other Austrian universities. The Vienna General Hospital is currently, in 2022 and 2023, listed among the 30 best hospitals in the world [41, 42]. At present, there is culminant activity in the building line of the Medical University of Vienna: In November 2023 eight construction cranes were simultaneously in action (Fig. 2), three of them for the future building for precision medicine which will be named after Eric Kandel, Nobel Prize laureate (with Austrian roots) in 2000 [43].

In the context of the implementation of the new medical curriculum in 2003 (see Fig. 1), the dropout rate continuously fell from 60% to 13% and the majority of students became able to complete their studies in the minimum amount of time [44]. Both developments were also due to the entrance restrictions for beginners, which was possible for the first time for the 2005/2006 course. Recently, in 2023, the diploma program in human medicine of the Medical University of Vienna received accreditation by the World Federation for Medical Education [45]. This means that the university’s students and graduates will be able to study, work and conduct research in the USA without restrictions.

In any case, it can be concluded that the development of independent medical universities in Austria cannot be judged negatively, as many predicted during their founding. Whether a comparable development would have been possible if medical faculties would have remained part of the parent university is doubtful due to the inadequate technical and legal framework conditions that then existed and still exist today. Since the execution of the separation of medicine from its former parent universities there is no intention on any side to 1:1 reverse this process. This does not mean that no thoughts should be given to sustainable new models for cooperation or even unifications between Austrian universities or parts of them.
